# Vaccination with Lipid Core Peptides Fails to Induce Epitope-Specific T Cell Responses but Confers Non-Specific Protective Immunity in a Malaria Model

**DOI:** 10.1371/journal.pone.0040928

**Published:** 2012-08-24

**Authors:** Simon H. Apte, Penny L. Groves, Mariusz Skwarczynski, Yoshio Fujita, Chenghung Chang, Istvan Toth, Denise L. Doolan

**Affiliations:** 1 Infectious Diseases Programme, Queensland Institute of Medical Research, Herston, Queensland, Australia; 2 School of Chemistry and Molecular Biosciences, University of Queensland, St Lucia, Queensland, Australia; 3 School of Pharmacy, University of Queensland, St Lucia, Queensland, Australia; 4 School of Medicine, University of Queensland, Herston, Queensland, Australia; Federal University of São Paulo, Brazil

## Abstract

Vaccines against many pathogens for which conventional approaches have failed remain an unmet public health priority. Synthetic peptide-based vaccines offer an attractive alternative to whole protein and whole organism vaccines, particularly for complex pathogens that cause chronic infection. Previously, we have reported a promising lipid core peptide (LCP) vaccine delivery system that incorporates the antigen, carrier, and adjuvant in a single molecular entity. LCP vaccines have been used to deliver several peptide subunit-based vaccine candidates and induced high titre functional antibodies and protected against Group A streptococcus in mice. Herein, we have evaluated whether LCP constructs incorporating defined CD4^+^ and/or CD8^+^ T cell epitopes could induce epitope-specific T cell responses and protect against pathogen challenge in a rodent malaria model. We show that LCP vaccines failed to induce an expansion of antigen-specific CD8^+^ T cells following primary immunization or by boosting. We further demonstrated that the LCP vaccines induced a non-specific type 2 polarized cytokine response, rather than an epitope-specific canonical CD8^+^ T cell type 1 response. Cytotoxic responses of unknown specificity were also induced. These non-specific responses were able to protect against parasite challenge. These data demonstrate that vaccination with lipid core peptides fails to induce canonical epitope-specific T cell responses, at least in our rodent model, but can nonetheless confer non-specific protective immunity against *Plasmodium* parasite challenge.

## Introduction

Vaccines are one of the most cost effective and efficient health care interventions for the prevention of infectious diseases. Almost all licensed vaccines are based on the delivery of live, attenuated, or killed whole pathogens. Vaccines which contain the minimal microbial components necessary to stimulate appropriate immune responses are referred to as subunit vaccines. Subunit vaccines have a range of advantages over the use of whole pathogenic microorganisms, including: improved stability, reduced risk of autoimmunity and allergic responses, no risk of reversion to the virulent form, ability to direct immune responses towards a specified antigen or epitope, and capacity for large-scale production under good manufacturing conditions [1], [2]. Recombinant protein-based subunit vaccines have been widely evaluated in many disease systems, including malaria [3]. However, the leading asexual blood-stage and liver-stage recombinant protein subunit vaccines candidates against malaria (MSP1, AMA1 and LSA1) have all failed in recent phase 2a experimental challenge studies and phase 2b field trials [4] despite induction of high antibody titre, growth inhibitory activity, and CD4^+^ T cell responses. Such failures highlight the need for a redirection of subunit vaccine approaches.

Synthetic peptide-based vaccines offer many advantages over whole-organism vaccines due their amenability to large-scale production, their well-defined composition and purity, and their suitability for freeze-drying which eliminates the need for the cold-chain. Further advantages of epitope-based vaccines over current vaccines include increased potency and other qualitative aspects of the immune response, particularly when compared to the use of whole antigens. Epitope-based immunization has been shown to be effective in eliciting responses against multiple B cell, CD4^+^ T cell or CD8^+^ T cell epitopes, including subdominant CD8^+^ T cell epitopes [5]–[13]. Most importantly, the epitope approach has been used successfully to treat and/or prevent different types of disease in animal models, including acute or chronic viral infections [6], [7], [14], [15], parasitic and microbial infections [16], and cancer [17].

However, peptides have limited immunogenicity because the exclusion of other pathogen components often removes the “danger signal” [18] necessary to trigger an immune reaction. To overcome this problem an adjuvant is usually required for peptide-based subunit vaccine efficacy. Adjuvants based on aluminium salts remain the principal compounds licensed for human use [19]. However, aluminium adjuvants are quite weak immune stimulants, unstable when freeze-dried, and possess some toxicity. In contrast, highly efficient adjuvants used experimentally in animal models or for veterinary use are often toxic and are therefore unsuitable for human use. Moreover, there are currently no adjuvants licensed for human use that were designed to specifically enhance cell mediated immune responses; critical for the control of many pathogens, including intracellular parasites such as *Plasmodium* [20]. These concerns have prompted us to develop self-adjuvanting lipopeptide vaccine delivery systems [21], [22], [23]. The Lipid Core Peptide (LCP) incorporates a lipoamino acid-based non-microbial lipidic adjuvant with a poly-lysine multiple antigenic peptide (MAP) system [24] which allow the conjugation of multiple copies of peptide antigens. In contrast to palmitoyl-conjugated lipopeptides [25], [26], [27], [28], the LCP delivery system incorporates three major persistent components: a) a non-microbial lipopeptide moiety (composed of synthetic lipidic amino acids (LAAs)), which may be arranged into the peptide sequence with a glycine spacer; b) a branching moiety (usually based on polylysine); and c) appropriate peptide epitopes [29]. The lipidic self-adjuvanting moiety can be easily modified in terms of the presence of a spacer, the number of LAAs, and the length of their alkyl chains. The lysine branching allows the advantageous use of a MAP system. The level of the branching can be adjusted to suit the requirements. The lysine carrier permits the conjugation of multiple copies of the same peptide epitope as well as conjugation of many different peptide antigens [30]. It has been shown that the number of LAA and the length of alkyne chain of each LAA can control induction of antibody production. To this end, an optimal LCP structure for vaccine delivery has been defined [23]. Studies have reported that the LCP core (lipidic part of LCP) does not induce immune responses, with or without “irrelevant” peptide [23], [31] and that a physical mixture (in contrast to conjugation) of peptide epitope and lipidic part of the LCP does not stimulate any immune responses [31].

The LCP-based constructs can be stored in a freeze-dried form at room temperature and are stable to a wide range of peptidases. Lipid core peptide synthesis can be achieved using classical solid-phase peptide synthesis with only a single purification after the cleavage of the resultant peptide from the solid support. The physicochemical and immunological properties of LCP systems can be readily altered by changing a nature and number of lipoamino acids, degree of poly-lysine branching, and by attachment of targeting moieties such as carbohydrates [32], [33]. This system can also incorporate two or more different epitopes attached to the one carrier molecule [31].

LCP vaccines have proved effective inducers of antibody responses in animal models of *Chlamydia* and Group A streptococcus [31], [34]–[36] but, despite the importance of T cells for control of many infectious diseases, their capacity to induce robust CD4^+^ or CD8^+^ T cell responses has not yet been established [20]. Furthermore, CD4^+^ T cell help may be required for optimal CD8^+^ T cell activity [37], [38] although this requirement is not absolute [39]–[41]. Thus, vaccines are usually designed to include either pathogen-specific CD4^+^ T cell helper epitopes (as in the case of full-length or partial length recombinant protein subunit vaccines) or “universal” (promiscuous) CD4^+^ T helper epitopes such as PADRE [42], [43]. For diseases where vaccine-induced immune responses may be boosted by natural exposure, such as malaria, inclusion of pathogen-specific CD4^+^ T cell epitopes is desired. For *Plasmodium*, CD4^+^ and CD8^+^ T cell epitopes typically map to similar or overlapping regions [44], [45].

The *Plasmodium yoelii* rodent model of malaria is an ideal system in which to evaluate the potential of LCP T cell epitope-based vaccine constructs. Malaria, caused by infection with parasites of the genus *Plasmodium*, remains a significant public health problem with approximately half of the world's population at risk of the disease [46], [47]. The development of a malaria vaccine remains a high global health priority. The feasibility of developing a malaria vaccine is supported by data showing that sterile infection-blocking immunity can be achieved by experimental immunization with radiation-attenuated *Plasmodium* spp. sporozoites in mice and humans (reviewed in [48]) or by immunization with infectious sporozoites under the cover of drug prophylaxis [49]–[51]. Studies in animal models have implicated CD8^+^ T cells as critical effector cells in this protection (based on *in vivo* depletion, reconstitution, and adoptive transfer studies), and CD4^+^ T cells have also been implicated with an important role in both the induction and effector phases (reviewed in [52]). IFN-γ has been identified as a critical mediator of the irradiated sporozoite induced protection [53], [54].

A CD8^+^ T cell epitope on the sporozoite coat protein of *P. yoelii*, the circumsporozoite protein (PyCSP amino acids 280–288, sequence SYVPSAEQI), has been identified as the target of CD8^+^ cytotoxic T lymphocytes (CTL) that can eliminate infected hepatocytes from *in vitro* culture in an antigen-specific and genetically-restricted manner [55], [56]. Furthermore, *in vivo* adoptive transfer of CD8^+^ CTL against this epitope can protect against sporozoite-induced malaria in the absence of other parasite-specific immune responses [57], [58]. This epitope is the immunodominant epitope recognized by BALB/c (H-2^d^) mice immunized with radiation attenuated paasites, recombinant viral constructs, or plasmid DNA encoding PyCSP [55], [57]–[64] and *in vitro* CTL activity and IFN-γ production specific for this epitope correlate with protective immunity [61]. This CD8^+^ T cell epitope is nested within a dominant CD4^+^ T cell epitope (amino acids 280–295, sequence SYVPSAEQILEFVKQI). Another dominant CD4^+^ T cell epitope(s) has also been identified on PyCSP (amino acids 57–70, sequence KIYNRNIVNRLLGD) and immunization of mice with a multiple antigenic peptide based on this epitope induced proliferative T cell responses, CTL capable of eliminating infected hepatocytes *in vitro*, and conferred partial protection against sporozoite challenge [65]. The CTL response was specific for a subdominant CD8^+^ T cell epitope mapped to residues 58–67 (sequence IYNRNIVNRL) that is recognized following immunization with the multiple antigen peptide [65] but not in the context of whole CSP immunization after immunization with either the whole organism (irradiated sporozoite) or whole antigen (plasmid DNA). Immunization with a synthetic peptide corresponding to the partially overlapping Py1 epitope (amino acids 59–79, sequence (YNRNIVNRLLGDALNGKPEEK)) [66] primed CD4^+^ T cells as well as CD8^+^ T cells which could eliminate parasites from infected hepatocytes *in vitro* [63]. This CD8+ T cell response is likely directed against the 9 mer subdominant CD8^+^ T cell epitope (residues 59–67, sequence YNRNIVNRL) [65]. Accordingly, we have evaluated the immunogenicity and protective capacity of LCP-based constructs expressing different combinations of these four well defined CD8^+^ and/or CD4^+^ T cell epitopes from PyCSP (Table 1).

**Table 1:** CD4^+^ and CD8^+^ T cell epitopes from *P. yoelii* circumsporozoite protein (*Py*CSP).

Peptide	Origin	mer	Amino acid sequence	*K*d IC50 (nM) [91]	Epitope characteristics	Ref
**P1**	*Py*CSP_280–288_	9	SYVPSAEQI	2.2	Immunodominant CD8	[55], [56]
**P2**	*Py*CSP_280–295_	16	SYVPSAEQILEFVKQI	463	CD4 dominant/CD8 dominant	[55]
**P3**	*Py*CSP_58–67_	10	IYNRNIVNRL	16	subdominant CD8	[65]
**P4**	*Py*CSP_57–70_	14	KIYNRNIVNRLLGD	8,250	CD4 dominant/CD8 subdominant	[65], [92]

## Materials and Methods

### Synthetic peptides and plasmid DNA

Synthetic peptides representing each of the defined CD4^+^ and/or CD8^+^ T cell epitopes from PyCSP were synthesized commercially (Mimotopes) at >80% purity. Plasmid DNA encoding full-length PyCSP, based on the VR1020 backbone, has been described previously [67].

### Design and Synthesis of the Lipid core peptide (LCP) Vaccines

**Figure 1:**
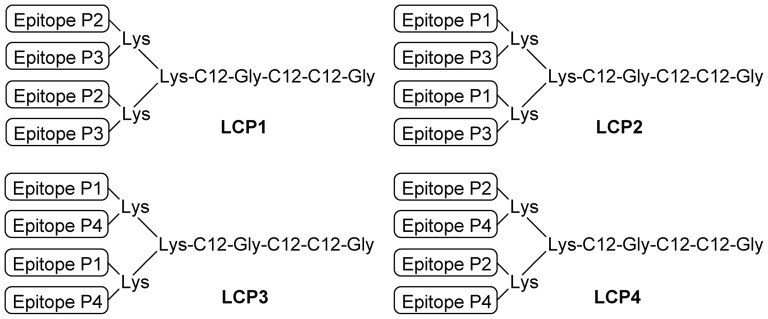
Schematic representation of lipid core peptide (LCP) constructs. Epitopes from *P. yoelii* circumsporozoite protein (*Py*CSP) as follows: P1 = *Py*CSP_280–288_, SYVPSAEQI; P2 = *Py*CSP_280–295_, SYVPSAEQILEFVKQI; P3 = *Py*CSP_58–67_, IYNRNIVNRL; P4 = *Py*CSP_57–70_, KIYNRNIVNRLLGD. See *Materials and Methods* and *Table 1* for additional details.

Lipid core peptide (LCP) vaccines were prepared by solid-phase peptide synthesis according to previously reported procedures [36], [68] at 20–40 mg each. Four LCPs were constructed (Figure 1). LCP1 contained two copies each of the 9 mer CD8^+^ T cell dominant epitope nested within a dominant CD4^+^ T cell epitope (280–288) and the 10 mer subdominant CD8^+^ T cell epitope (58–67). LCP2 contained two copies each of the 9 mer CD8^+^ T cell dominant epitope (280–288) and the 10 mer subdominant CD8^+^ T cell epitope (58–67). LCP3 contained two copies each of the 9 mer CD8^+^ T cell dominant epitope (280–288) and the 10 mer subdominant CD8^+^ T cell epitope nested within a dominant CD4^+^ T cell epitope (55–70). LCP4 contained two copies each of the two dominant CD4^+^ T cell epitopes (57–70 and 280–295).

### Haemolytic assay

Since LCP compounds incorporate hydrophobic lipid moieties and hydrophilic peptide epitopes, they have amphiphilic (surfactant-like) properties. Therefore the capacity of the LCP compounds to induce haemolysis was examined in a standard haemolytic assay. In brief, blood collected from a healthy human volunteer with written informed consent (protocol approved by the University of Queensland Ethics Committee, approval number 2009000661) was centrifuged at 750 *g* for 15 min and washed with PBS (Gibco-BRL) until the suspension was transparent. The red blood cell pellet was resuspended to original volume with PBS and 100 µL of red blood cell solution added per well in a 96-well plate (Greiner CELLSTAR®). Subsequently, 100 µL of each LCP construct at 10 µM, 50 µM, and 200 µM concentration was added to triplet wells and incubated at 37°C for 1 h. SDS and PBS control treatments were assayed in parallel. After 1 h, the plate was centrifuged at 750 *g* for 15 min and then 75 µL of supernatant per well was transferred to a new 96-well plate and measured by UV spectrometer at 540 nm wavelength. The percentage of hemolysis was evaluated by comparing the absorbance (540 nm) of the vaccine candidates with that of negative control (SDS, 100% hemolysis) and positive control (PBS, 0%). Data were analysed according to the following formula:
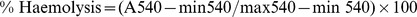



It was clearly demonstrated that the LCP constructs are not haemolytic even at high concentration (200 µM) (Figure S1).

### Animals and Parasites

Specific pathogen-free female BALB/c mice (Animal Resources Centre, Perth, Australia) were used at 6–9 weeks of age. Female CS-TCR transgenic mice (BALB/c background) were kindly provided by Prof. Fidel Zavala (Johns Hopkins University, Baltimore, USA); the CS-TCR mouse has been engineered so that the TCR is specific for the immunodominant CD8^+^ T cell epitope from *Plasmodium yoelii* CSP (residues 280–288) [69]. C57BL/6 RAG-2^−/−^γc^−/−^ were bred at QIMR. All studies were approved by the QIMR Animal Ethics Committee and were conducted in accordance with the Australian Code of Practice for the Care and Use of Animals for Scientific Purposes (2004).

### Immunizations and adoptive transfer studies

Mice were immunized subcutaneously (s.c.) one to three times with 30 µg LCP at 3 week intervals and assessed for immunogenicity or protective efficacy against *Plasmodium yoelii* sporozoite challenge at 11–14 days post final immunization. For the former, mice were sacrificed and splenocytes and lymph nodes were harvested, processed and assayed for phenotypic markers or cytokine response. In some studies, CD8^+^ T cells (50,000) purified from naïve-CS-TCR transgenic mice were adoptively transferred via intravenous (i.v.) injection into the tail vein of naïve BALB/c mice 2 days prior to LCP immunization. At 7 days post vaccination, splenocytes and draining lymph nodes were harvested and the presence and function of donor CD8^+^ T cells were analysed by flow cytometry. Alternatively, mice were immunized s.c. with matched doses of synthetic peptides in CpG/alum (CpG ODN 1826, 50 µg/dose (Sigma Aldrich) mixed 1∶1 with Alhydrogel® (Brenntag Biosector, Frederikssund, Denmark) given in a 200 µl dose) or intramuscularly (i.m.) in each tibialis anterior muscle with 50 µg of PyCSP plasmid DNA.

### Flow cytometry and analysis

Cells were incubated on ice with combinations of fluorochrome-conjugated Ab to CD8+ells were incubated on ice with combinations of fluorochrome-conjugated Ab to CD8e presence and function of donor CD8ice dies, ice for the Care and Use of Animals with 1 mg/ml propidium iodide (Calbiochem, San Diego, CA). CD8^+^ T cells were purified using a MoFlo cytometer (DakoCytomation, Glostrup, Denmark) with exclusion of dead cells based on forward scatter and propidium iodide uptake. Flow cytometric analysis was performed on a FACSCalibur (BD Biosciences, North Ryde, NSW, Australia) or FACSCanto II (BD Biosciences) with standard optics configuration (405 nm violet laser, 488 nm blue laser, 633 nm red laser) with CellQuest version 3.1F software (BD Biosciences, San Jose, CA). Post-acquisition data analysis was performed with FlowJo software version 9.1 (Treestar, Ashland, OR, USA) or Summit Software V4.3 (DakoCytomation). Calculations were performed using Microsoft Excel (version 12, Microsoft Corporation, WA, USA) or Prism GraphPad software V5.0 (La Jolla, CA, USA).

### Cytometric bead array

Splenocytes from vaccinated mice were cultured with each of the synthetic peptides representing the defined CD8^+^ or CD4^+^ T cell epitopes, ConA, or media only, for three days. Culture supernatants from individual mice were collected and cytokines were analysed according to the manufacturer's instructions using the cytometric bead array (CBA) (BD Biosciences). Analysis was performed on a FACSarray cytometer equipped with CellQuest Pro and CBA software (BD Biosciences).

### CTL assay

To assess cytotoxic capacity, splenocytes harvested seven days post-vaccination were restimulated for three days with synthetic peptides representing the dominant CD8^+^ T cell epitope (P1; CSP_280–288_). To assess cytolytic function the splenocytes were incubated for 4–5 hours at the ratio of 100∶1 with CFSE stained target cells of the A20 cell line that had been pre-incubated for 60 minutes with the P1 peptide or anti-receptor monoclonal antibody 2C11. Cytolytic function was determined by flow cytometric assessment of the percentage of CFSE labeled target cells that were propidium iodide positive at the completion of the incubation, as described previously [70].

### Flow-based antibody assay

Serum antibody levels were determined by flow cytometric evaluation of median fluorescence intensity (MFI) of parasite extract incubated with serum and then stained with fluorescently labelled secondary antibodies to pan-Ig or specific isotypes detailed below.

For preparation of extract, *P. yoelii* YM was grown in RAG-2^−/−^ γc^−/−^ mice (deficient in T, B, and NK cells) to approximately 75% parasitemia. Blood was collected from anesthetized mice by cardiac puncture into 10 volumes of FCAB buffer [71] prepared from PBS with 2 mM EDTA and 0.5% heat inactivated FBS stock solutions (Sigma–Aldrich, Castle Hill, NSW, Australia). Samples were centrifuged at 600 RCF for 10 minutes, aspirated and resuspended in 0.5% w/v saponin, and incubated for 30 minutes at 37°C. The sample was drawn twice through a 30 gauge needle before being washed twice with 20 volumes of Milli-Q H_2_0 and centrifuged as above. The sample was then fixed in FCAB fixation and lysis buffer [71] for 10 minutes at 37°C. FCAB fixation and lysis buffer prepared from PBS with 4% w/v paraformaldehyde and 0.0067% w/v saponin (both Sigma-Aldrich). The extract was resuspended in FCAB buffer and stored at −20°C until required. All buffers were sterile filtered (0.2 µm) before use.

For the assay, 3 µl blood samples were collected and diluted in 1 ml of FCAB buffer as described [71]. Serum supernatant from centrifuged blood samples was incubated with extract for 10 minutes at 4°C in 96-well v-bottom plates, washed with three volumes of FCAB buffer and then stained with a cocktail of anti-Ig antibodies for ten minutes at 4°C: anti-IgG2a-FITC (clone R19-15), anti-IgM-PE/Cy7 (clone R6-60.2), anti-IgG1-APC (clone A85-1), and GAM-FITC (poly 554001) (all BD Biosciences); and anti-IgE-PE (clone RME-1) and GAM-APC/Cy7 (poly 4053) (both Biolegend). Following another wash, the samples were resuspended in 35 µl of FCAB buffer and analysed on FACSCanto II (BD Biosciences, North Ryde, NSW, Australia) equipped with HTS plate reader. Post-acquisition data analysis was performed with FlowJo software version 9.1 (Treestar, Ashland, OR, USA); calculations were performed using Microsoft Excel (version 12, Microsoft Corporation, WA, USA). Relative Ab fluorescence intensity was determined by dividing the MFI of the sample serum by the mean MFI of serum samples from five naïve mice at each time point.

### In vivo protection

Mice were challenged by tail-vein injection with 1000 cryopreserved infectious *P. yoelii* sporozoites (17XNL non-lethal strain) (Sanaria Inc., Rockville, MD, USA). For evaluation of partial protection at the liver-stage, mice were euthanized at 42 hours after challenge and the liver-stage parasite burden assessed using an assay modified from that described previously [72]. Briefly, the livers were collected and homogenized in RLT Buffer (Qiagen) and aliquots of liver RNA extracted using RNAeasy® Mini Kits (Qiagen). cDNA was synthesised using SuperScript VILO cDNA Synthesis Kit (Invitrogen). Parasite RNA was extracted and *P. yoelii* 18S ribosomal RNA quantified by quantitative real time PCR (Py18S 5′ primer Py685F 5′-CTTGGCTCCGCCTCGATAT; Py18S 3′ primer Py782R 5′-TCAAAGTAACGAGAGCCCAATG; Py18S probe 6FAM-CTGGCCCTTTGAGAGCCCACTGATT-BHQ-1); β2-microglobulin was quantified using TaqMan®. An estimate of *P. yoelii* 18S rRNA “plasmid equivalents” and mouse β2-microglobulin (housekeeping gene) “plasmid equivalents” were derived from the Ct (Threshold Cycle) measured for each PCR target for each unknown sample. Quantitative parasite burden data was expressed as the ratio of *P. yoelii* 18S rRNA plasmid equivalents over the mouse β2-microglobulin plasmid equivalents for each sample. Alternatively, sporozoite-infected mice were allowed to progress to blood-stage parasitemia or were challenged with 1×10^5^ *P. yoelii* blood-stage parasites, and the course of infection was monitored by a recently described multiparameter flow cytometry assay (FCAB assay) up to day 35 post-challenge [71].

### Statistical analyses

Assessment of statistical significance was performed using one-way ANOVA with Bonferroni's post-hoc test (Prism 4.02 software package; Graph-Pad, San Diego, CA). Significance was defined at the 5% level. Values of *p* are indicated in the figures by the following symbols: NS, not significant, *p*>.0.05; * *p* = 0.01–0.05; ** *p* = 0.001–0.01; *** *p*<0.001.

## Results

### PyCSP epitope-based LCP vaccines fail to induce antigen-specific CD8^+^ T cell expansion

Initially we determined whether our lipid peptide core (LCP) vaccines could induce the expansion of PyCSP-specific CD8^+^ T cells by adoptively transferring naïve Vβ8.1^+^ Thy1.1^+^ CD8^+^ T cells purified from CSP_280–288_-specific TCR-transgenic (CS-TCR) mice into WT BALB/c hosts. Two days later the mice were vaccinated s.c. with individual LCP (LCP1, LCP2, LCP3, or LCP4), or a pool of cognate peptides representing the target T cell epitopes formulated in CPG/alum (peptides 1–4, see Table 1 above), or i.m. with DNA vaccine encoding the *Py*CSP. Seven days post vaccination, draining lymph nodes (DLN) and spleens were harvested and donor cell presence assessed by flow cytometry. Preliminary experiments first determined the optimal number of cells for adoptive transfer to be 50,000 (data not shown) and this was used in all subsequent experiments involving adoptive transfer. The percentage of the CD8^+^ T cell population consisting of donor cells was not increased in the spleen or DLN after a single immunization with any vaccine.

**Figure 2:**
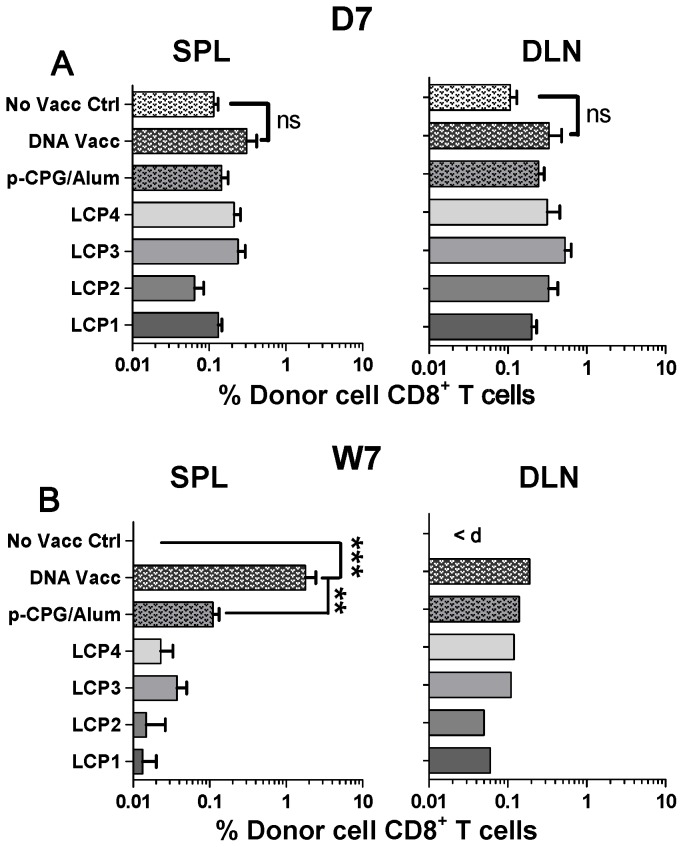
Antigen-specific donor CD8^+^ T cell expansion following vaccination. Frequency of CS-TCR CD8^+^ T cells (with specificity for the dominant CD8^+^ T cell epitope from *P. yoelii* circumsporozoite protein, *Py*CSP_280–288_) within the draining lymph nodes (DLN) and spleen (SPL) seven days after adoptive transfer into congenic hosts and vaccination with a single s.c. injection of lipid core peptides (LCP), or pooled peptides (P1, P2, P3, P4; see *Table 1*) and CpG-Alum, or i.m. injection with DNA vaccine encoding *Py*CSP (D7) (A); or seven days after the final of three similar vaccinations given at 3 week intervals (W7) (B). Results (mean and SEM) with mice tested individually are shown (A, SPL and DLN n = 6 mice pooled data from two repeat experiments; B, SPL n = 5 mice from a representative experiment) except for B, DLN which were pooled from five mice (mean shown). Statistical comparisons are made to a control group that received adoptively transferred cells but no vaccination (No Vacc Ctrl) or as indicated on the graph, using one-way ANOVA with Bonferroni's post-test.

To determine whether the expansion in antigen-specific CD8^+^ T cells could be enhanced by boosting, we adoptively transferred naïve CS-TCR CD8^+^ T cells into naïve BALB/c hosts and vaccinated mice as described above. Mice were then boosted at three and six weeks after the initial vaccination. Seven days following the third vaccination, the frequency of donor cells within the spleens of DNA vaccinated mice was significantly increased when compared to the no-vaccine control and other vaccine groups and constituted approximately two percent of the entire CD8^+^ T cell population (Fig. 2B), representing a near 10-fold enhancement over levels following the primary vaccination (Fig. 2A); a 2-fold enhancement was noted in the DLN. With peptide-CpG/alum, CD8^+^ T cells were maintained at similar levels in both spleen and DLN. In contrast, there was at least a 10-fold reduction in CD8^+^ T cells in the spleen and 3–5 fold reduction in CD8^+^ T cells in the DLN following LCP vaccinations. Donor cells were no longer detectable in the spleens or DLN of unvaccinated control mice at this time point.

### LCP vaccines induce a non-specific type 2 polarized response rather than a canonical CD8^+^ T cell type 1 response

**Figure 3:**
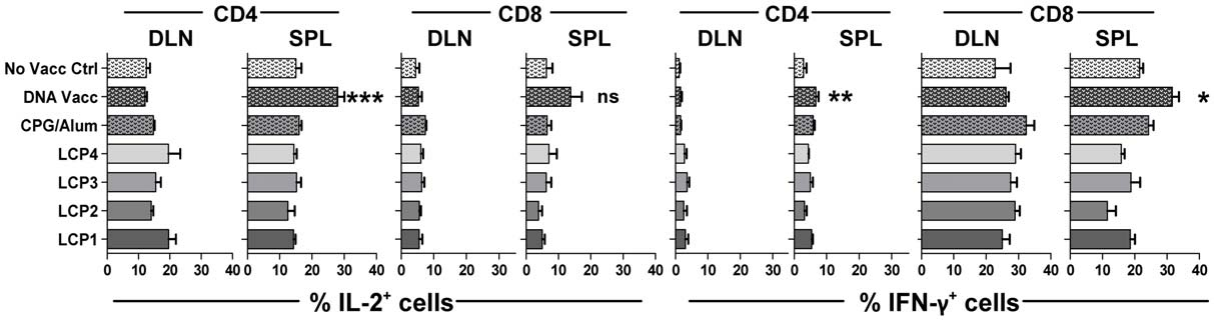
IL-2 and IFN-γ expressing T cells following vaccination. Frequency of CD4^+^ and CD8^+^ T cells expressing IL-2 and IFN-γ following PMA/ionomycin restimulation and intracellular staining of cells taken from the draining lymph nodes (DLN) and spleen (SPL) seven days after vaccination with a single s.c. injection of lipid core peptides (LCP), or pooled peptides (P1, P2, P3, P4; see *Table 1*) and CpG-Alum, or i.m. injection with DNA vaccine encoding *Py*CSP. Results pooled from two repeat experiments are shown (n = 6 mice, mean and SEM). Statistical comparisons are made to a control group that received no vaccination (No Vacc Ctrl) using one-way ANOVA with Bonferroni's post-test.

To determine the effects of LCP vaccination on the functional phenotype of the responding T cells, mice were vaccinated with LCP vaccines or controls including p-CPG/alum or DNA vaccine in a single or prime-boost strategy as described above. Seven days after primary vaccination, IFN-γ and IL-2 expression was assessed by ICS following a 4.5 h PMA/ionomycin restimulation of T cells recovered from spleens and DLNs (Fig. 3). DNA vaccination significantly increased the frequency of CD8^+^ T cells and CD4^+^ T cells producing IFN-γ the spleen but not DLN and the frequency of IL-2 producing CD4^+^ cells in the spleen. None of the other vaccines had a consistent effect on T cell cytokine expression.

**Figure 4:**
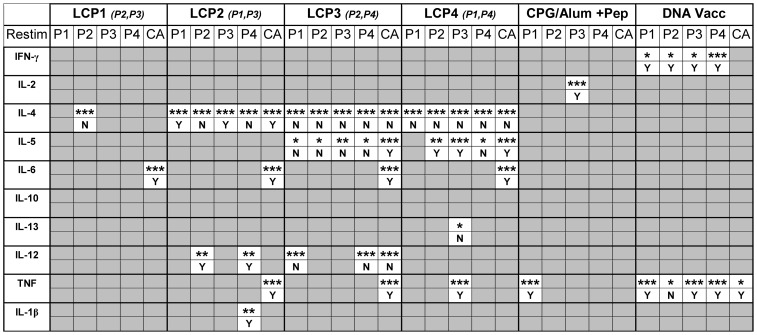
Cytokine production following prime-boost vaccinations. Mice were vaccinated s.c. three times at three week intervals with lipid core peptides (LCP), or pooled peptides (P1, P2, P3, P4; see *Table 1*) and CpG-Alum, or i.m. injection with DNA vaccine encoding *Py*CSP. Seven days following the final vaccination the splenocytes were harvested and restimulated for three days with peptides as indicated. Cytokine levels in the supernatant were then determined by Cytokine Bead Array. Asterisks indicate a significant increase in cytokine production when compared to the restimulation-equivalent no-vaccine control. Y (yes) or N (no) indicate statistically significant (p = <0.05) increase in cytokine production when compared to those observed in controls that had received the same vaccinations but were cultured without peptide or ConA (media only). Significance determined using one-way ANOVA with Bonferroni's post-test (n = 5).

In parallel experiments, splenocytes harvested seven days following the final prime-boost vaccination were cultured with individual PyCSP peptides, or controls including ConA or media only, for three days. Cytokine levels in the supernatant were determined by Cytokine Bead Array (CBA) for a panel of cytokines (Fig. 4). We first assessed whether vaccination had affected the cytokine levels by comparing levels to the restimulation-equivalent no-vaccine control (shown as asterisks when significant). We then assessed whether any changes in cytokine responses were peptide-specific by comparing levels to those observed in controls that had received the same vaccinations but were cultured without peptide or ConA (media only) (shown as Yes (Y) or No (N); Yes indicating p<0.05).

In contrast to DNA, and consistent with the ICS data, LCP vaccination failed to induce epitope-specific production of IFN-γ (Fig. 4). Rather, based upon the CBA data, the LCP vaccines induced a predominantly type-2 polarized cytokine response, but these responses were almost exclusively not epitope-specific.

**Figure 5:**
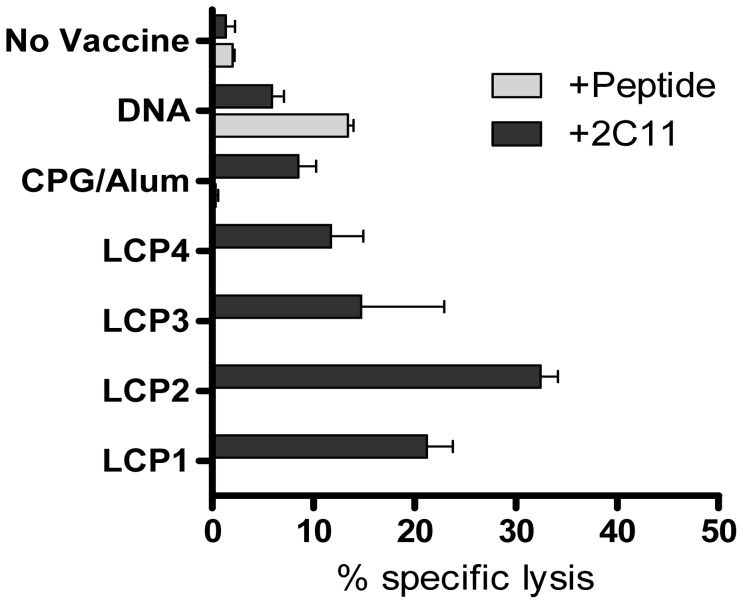
Antigen-specific CTL function following prime-boost vaccination with Lipid Core Peptides. Mice were vaccinated s.c. three times at three week intervals with lipid core peptides (LCP), or pooled peptides (P1, P2, P3, P4; see *Table 1*) and CpG-Alum, or i.m. injection with DNA vaccine encoding *Py*CSP. Seven days after the final vaccination the splenocytes were harvested and restimulated for three days with the dominant CD8^+^ T cell epitope (Pep 1, *Py*CSP_280–288_) and then assessed for cytolytic potential by flow cytometry using A20 target cells coated with Pep 1 (+Peptide) or anti-receptor antibodies (+2C11) (see *Materials and Methods*). Results from a representative experiment are shown (triplicate samples, mean and SEM).

To further investigate the functional ability of LCP vaccine responding CD8^+^ T cells, a CTL assay was performed with splenocytes harvested seven days after the final vaccination from prime-boost vaccinated mice. Splenocytes from prime-boost LCP-vaccinated mice failed to kill peptide-coated target cells, but were able to kill anti-receptor Ab coated target cells (Fig. 5). These data suggest that the LCP vaccines induced CTL responses of unknown specificity.

Taken together, data show that the LCP vaccines did not induce a canonical CD8^+^ T cell type 1 response directed against the epitopes included in the constructs, but rather a non-specific type 2 polarized response.

### LCP vaccination modifies the antibody response to infection

**Figure 6:**
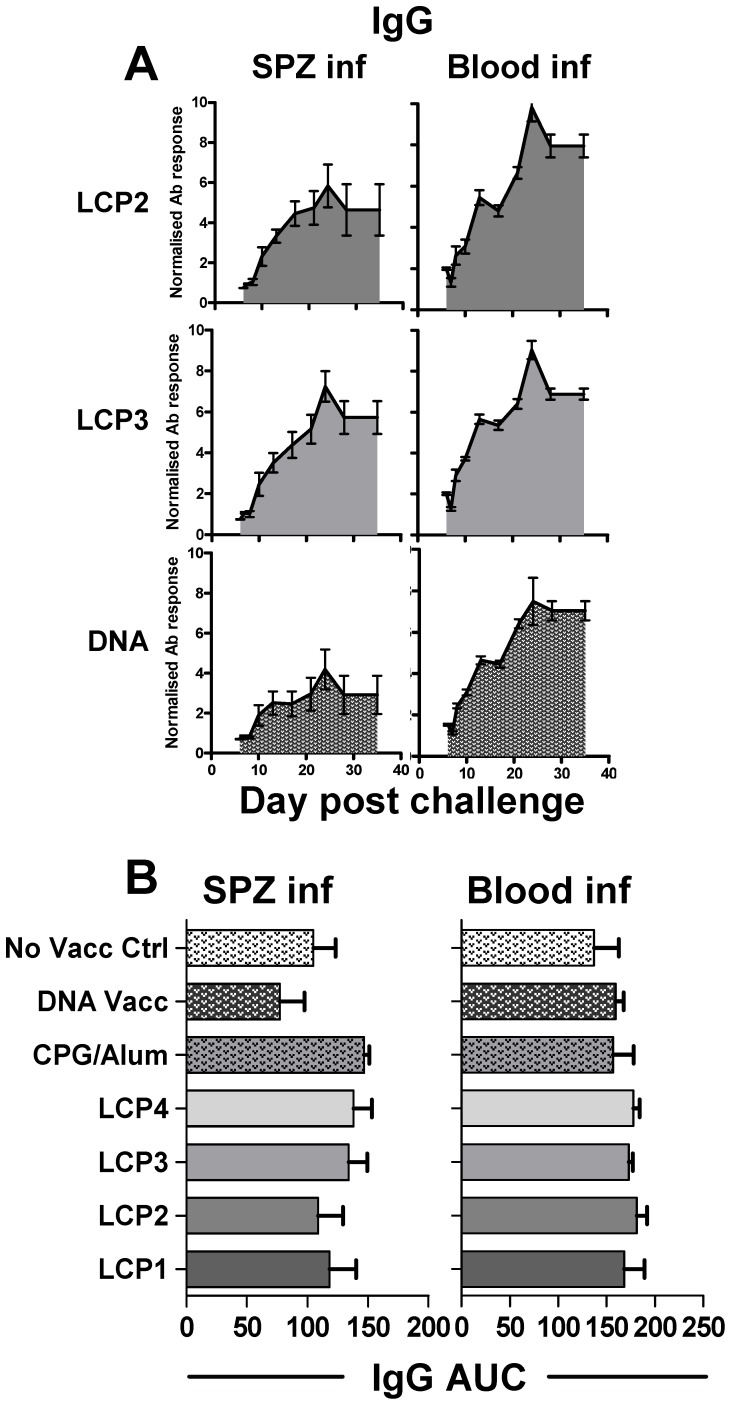
Parasite-specific IgG response following vaccination and infection. Mice were vaccinated s.c. three times at 3 week intervals with lipid core peptides (LCP), or pooled peptides (P1, P2, P3, P4; see *Table 1*) and CpG-Alum, or i.m. injection with a DNA vaccine encoding *Py*CSP. Seven days after the final vaccination the mice were challenged by i.v. injection with live *P. yoelii* sporozoites (SPZ inf) or parasitized red blood cells (Blood inf). The parasite-specific IgG antibody response was measured during the course of infection by flow cytometry (see *Materials and Methods*) and the area under the curve (AUC) for each individual mouse calculated. (A) Representative plots demonstrating the kinetics of the IgG response and the AUC. (B) Statistical comparisons for all groups. Results from a representative experiment are shown (n = 7 mice, mean and SEM). Statistical comparisons were made to a control group that received no vaccination (No Vacc Ctrl) using one-way ANOVA with Bonferroni's post-test (no significant differences noted).

Given the apparent Th2 cytokine polarization induced by LCP vaccination, we hypothesized that the antibody response induced by the LCP constructs may be distinct from that induced by plasmid DNA. Blood-stage parasite-specific antibody responses were undetectable following vaccination with PyCSP DNA (PyCSP is not expressed in the blood-stage of the parasite life cycle) but developed rapidly concomitant with patent parasitemia induced by either sporozoite or blood-stage parasite challenge. The kinetics of the parasite-specific IgG response of the different vaccine groups followed a similar pattern (Fig. 6*A* *- only LCP2, LCP3, and DNA Vaccine shown for simplicity*). No significant differences in the AUC of the IgG response were observed between the different vaccine groups and further assessmentof the relative amount of parasite-specific IgM, IgG1 and IgG2a also failed to show any significant differences (data not shown).

**Figure 7:**
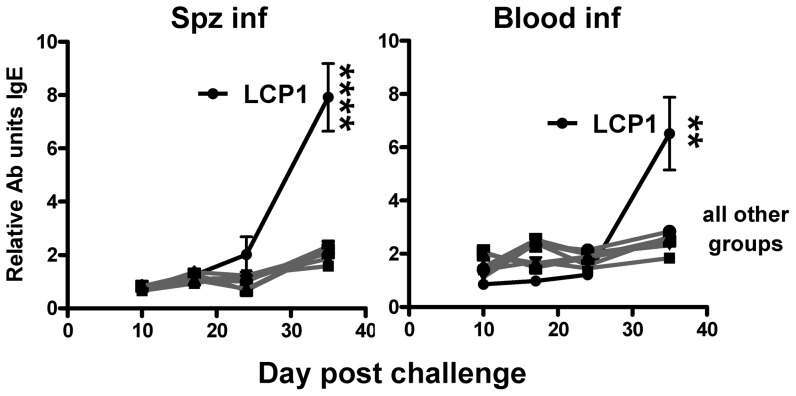
Parasite-specific IgE response following vaccination and infection. Mice were vaccinated s.c. three times at 3 week intervals with lipid core peptides (LCP), or pooled peptides (P1, P2, P3, P4; see *Table 1*) and CpG-Alum, or i.m. injection with DNA vaccine encoding *Py*CSP. Seven days after the final vaccination mice were challenged by i.v. injection with live *P. yoelii* sporozoites (SPZ inf) or parasitized red blood cells (Blood inf). The parasite-specific IgE response was measured at the timepoints indicated during the course of infection by flow cytometry (see *Materials and Methods*). Results from a representative experiment are shown; statistical comparisons are made between the LCP1 group and a similarly infected control group that was not vaccinated using one-way ANOVA with Bonferroni's post-test (n = 7 mice, mean and SEM).

Strikingly, a marked increase in parasite-specific IgE levels was observed following the resolution of parasitemia in the LCP1 vaccine groups in mice infected with either sporozoites or with infected RBC (Fig. 7). The generation of an IgE response is a natural conclusion to a Th2 characterised response [73], [74]; curiously, however, this IgE response was not pronounced in mice immunized with the other LCP vaccines which were not markedly different from either CpG/alum or plasmid DNA immunized mice.

### LCP vaccines protect against experimental malarial challenge

**Figure 8:**
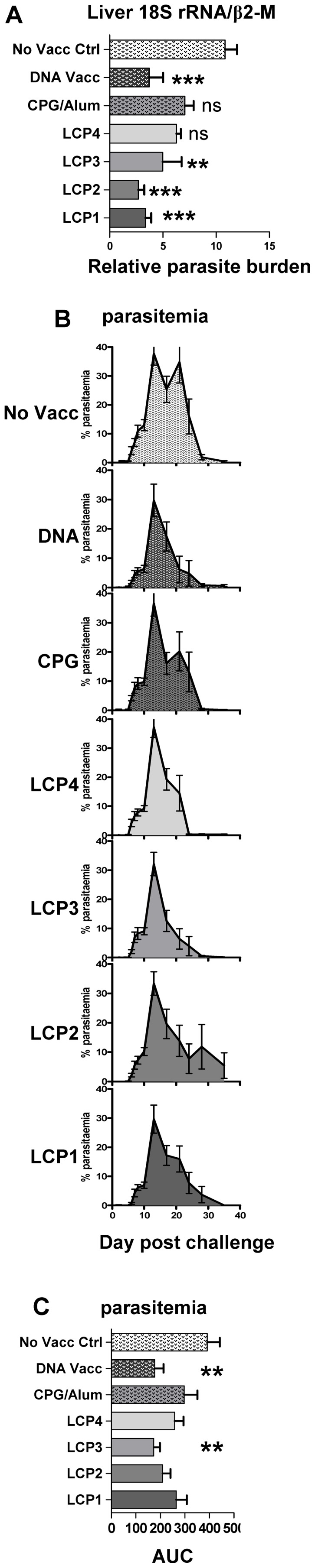
Parasite burden and parasitemia following vaccination and infection. Mice were vaccinated s.c. three times at three week intervals with lipid core peptides (LCP), or pooled peptides (P1, P2, P3, P4; see *Table 1*) and CpG-Alum, or i.m. injection with DNA vaccine encoding *Py*CSP. Seven days after the final vaccination mice were challenged by i.v. injection with live *P. yoelii* sporozoites (SPZ inf) or parasitized red blood cells (Blood inf). (A) Liver parasite burden in sporozoite infected mice, assessed 42 h post infection by RT-PCR of parasite 18S rRNA (see *Materials and Methods*) (n = 5 mice, mean and SEM). (B) Kinetics of parasitemia and (C) area under curve of parasitemia (AUC); results of pooled data from three repeat experiment are shown (n = 21 mice, mean and SEM). Statistical comparisons are made to a control group that received no vaccination (No Vacc Ctrl) using one-way ANOVA with Bonferroni's post-test.

Since the most appropriate assessment of a vaccine platform is capacity to protect against pathogen challenge, naïve mice were vaccinated with each of the four LCP vaccines or controls including p-CPG/alum or DNA encoding *P. yoelii* CSP in a prime-boost strategy as described above. Eleven days following the final vaccination, mice were challenged with 1000 *P. yoelii* sporozoites. Liver-stage parasite burden was determined 42 h post-challenge by RT-PCR of *P. yoelii* 18S ribosomal RNA (Fig. 8*A*). In parallel experiments, mice were allowed to progress to blood-stage parasitemia (Fig. 8*B*). The kinetics and burden of blood-stage infection were monitored to day 35 post-infection (Fig. 8*B*) and the ability of the vaccines to inhibit the development of blood-stage parasitemia was assessed by comparative area under the curve (AUC) analysis (Fig. 8*C*).

No mice were sterilely protected following immunization and sporozoite infection; however, consistent with previous studies [61], the PyCSP plasmid DNA vaccine conferred significant protection at the liver-stage as indicated by both significant reduction in liver-stage parasite burden (Fig. 8*A*) and blood-stage parasitemia following sporozoite challenge (Fig. 8*B and 8C*). LCP3 also conferred a significant reduction in liver-stage parasite burden and blood-stage parasitemia; whereas LCP1 and LCP2 conferred protection against liver-stage parasite burden only. Mice immunized with LCP4 or with the pool of CD8^+^ and CD4^+^ T cell epitopes formulated in CpG/alum were not protected at any stage.

## Discussion

In 1984, it was reported that conjugation of a dipalmityl-lysine moiety to a synthetic peptide derived from the hepatitis B surface antigen (HBsAg) significantly improved the anti-HepB antibody response, in comparison to the corresponding peptide-keyhole limpet hemocyanin conjugate [75]. Subsequently it was demonstrated that influenza virus-specific CD8^+^ CTL could be primed *in vivo* by a synthetic lipopeptide vaccine comprising synthetic peptide epitopes covalently linked to tripalmitoyl-S-glycerylcysteinyl-seryl-serine, in the absence of adjuvant, whereas the corresponding peptide without a lipidic moiety did not [76]–[78]. It is now well established that lipopeptides, in particular tripalmitoyl-S-glyceryl cysteine (Pam3Cys) lipopeptides, constitute potent immunoadjuvants in animal models, markedly enhancing the epitope-specific immune response when conjugated to B cell, helper T cell, or cytotoxic T lymphocyte epitopes; improving vaccine efficiency and conferring protection against pathogen challenge in animal models (reviewed in [5], [23], [79], including malaria [25]–[28]. Of particular importance for vaccine development is the ability of lipopeptides to induce CD8^+^ T cell and CTL responses (reviewed in [79]), which are key mediators of protection against intracellular pathogens. Lipopeptides are thought to induce dendritic cell (DC) maturation and production of pro-inflammatory cytokines (with a Th1-bias for palmitoyl-lipopeptides), activating antigen-specific CD4^+^ and CD8^+^ T cell responses via the Toll-like receptor-2 pathway [80].

Previously, we have reported a promising vaccine delivery system, lipid core peptide (LCP) [29], [81]. LCP is designed to incorporate antigen, carrier, and adjuvant in a single molecular entity. The mode of action of LCP-based vaccine candidates has been analysed and it was found that, similar to other lipopeptides, the self-adjuvanting activity of constructs was based on TLR2 mediated DC activation [82]–[84]. Studies have shown that incorporation of a monomeric peptide epitope into a LCP structure could enhanced antibody immunogenicity up to 3200-fold in a mouse model of *Chlamydia* [34] and that the magnitude and specificity of the LCP-induced antibody responses could be influenced by the number of epitope sequences attached to the oligomeric polylysine core, the number of lipoamino acids in the constructs, the length of the alkyl side-chains, or the spacing between the lipoamino acid units [33], [35]. LCP constructs incorporating defined B cell epitopes have induced highly opsonic antibodies and conferred protection against infection with Group A streptococcus in mice even in the absence of adjuvant [31], [36]. However, there are no reports of robust LCP-induced T cell responses. One study showed that an LCP construct consisting of four copies of a minimal CD8^+^ T cell epitope attached to a core containing lipoamino acids stimulated a cytotoxic CD8^+^ T-cell response *in vivo* but only in the presence of alum as an additional adjuvant [85].

As yet, there is no solid evidence that LCP constructs can access either MHC class I or class II presentation pathways to induce epitope-specific T cell responses and confer protection in a T cell dependent manner. CD4^+^ T cells recognize peptide epitopes derived from exogenous antigens taken up by antigen presenting cells, in association with MHC class II molecules. In contrast, antigens recognized by CD8^+^ T cells are generally processed by the endogenous pathway and presented to CD8^+^ T cells in association with MHC class I molecules [86]–[89]. For exogenous antigens to be presented in complex with MHC class I they must be cross-presented (reviewed in [90]) and the lipid moiety of lipopeptides appears to facilitate cross-presentation, enabling robust CD8^+^ T cell responses. However, since the structure of LCP constructs is more complex than that of lipopeptides, it is not obvious that LCPs would be similarly processed. Accordingly, the aim of this study was to determine whether LCP constructs incorporating defined CD4^+^ and/or CD8^+^ T cell epitopes, with or without specific CD4^+^ T cell help, could induce epitope-specific immune response and protect against pathogen challenge in a rodent model of malaria.

In the work presented herein, LCP vaccines failed to induce an expansion of antigen-specific CD8^+^ T cells following primary or prime-boost immunization. Furthermore, LCP vaccines induced type 2 polarized cytokine responses and cytotoxic responses that were not specifically directed against the antigen epitopes. This was in contrast to the responses elicited by DNA vaccination which was characterized by a canonical antigen-specific CD8^+^ T cell expansion and the production of IFN-γ and TNF. The non-specific LCP-induced responses were nonetheless able to protect against parasite challenge, since three of the four LCP vaccines were able to significantly reduce liver-stage parasite burden. The protective LCPs contained dominant and subdominant CD8^+^ T cell epitopes that were not nested within CD4^+^ T cell epitopes (a 9 mer and 10 mer), whereas the LCP containing dominant CD4^+^ T cell epitopes spanning those CD8^+^ T cell epitopes was not protective (a 16 mer and 14 mer). These findings may reflect an inability of the LCPs to find their way into the cross-presentation pathway or an inability of the antigen-presenting cell to cleave the longer peptides as the 9 mer and 10 mer could conceivably bind free MHC-I molecules without requiring these functions. This again contrasts with the DNA immunization which induced antigen-specific CD8^+^ T cell expansion, presumably via a mechanism where antigen-presenting cells cross-present protein expressed within muscle; although, plasmid DNA taken up directly, and expressed by antigen-presenting cells, could achieve the same outcome. Indeed, it remains unknown which cells are responsible for taking up and presenting the LCPs in our study and if they are capable of cross-presentation. Further studies will be required to understand the mechanism of antigen presentation and type 2 cytokine induction observed in our model and whether further modification of the LCP-core could promote antigen-specific CD8^+^ T cell responses.

## Supporting Information

Figure S1 **Hemolytic potential of lipid core peptides.** Hemolytic potential of lipid core peptides (LCP) was measured by comparing the absorbance (540 nm) of blood samples incubated with the LCP vaccine candidates with that of samples incubated with a positive control (SDS, 100% hemolysis) and a negative control (PBS, 0%) (see *Materials and Methods*). Mean and SD of triplicates samples shown.
(PDF)
