# Supplementary figures and images for: Vaccination with Lipid Core Peptides Fails to Induce Epitope-Specific T Cell Responses but Confers Non-Specific Protective Immunity in a Malaria Model
Source: PLoS One. 2012 Aug 24;7(8):e40928. doi: 10.1371/journal.pone.0040928 (PMC3427299; doi:10.1371/journal.pone.0040928)

**Supplementary Figure 1**

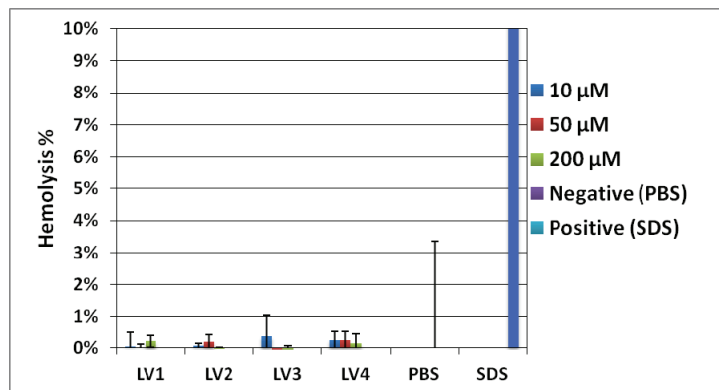

Supplement: Figure S1 — Hemolytic potential of lipid core peptides. Hemolytic potential of lipid core peptides (LCP) was measured by comparing the absorbance (540 nm) of blood samples incubated with the LCP vaccine candidates with that of samples incubated with a positive control (SDS, 100% hemolysis) and a negative control (PBS, 0%) (see Materials and Methods ). Mean and SD of triplicates samples shown. (PDF) [file pone.0040928.s001.pdf]
